# Association of Temporary Financial Assistance With Housing Stability Among US Veterans in the Supportive Services for Veteran Families Program

**DOI:** 10.1001/jamanetworkopen.2020.37047

**Published:** 2021-02-10

**Authors:** Richard E. Nelson, Thomas H. Byrne, Ying Suo, James Cook, Warren Pettey, Adi V. Gundlapalli, Tom Greene, Lillian Gelberg, Stefan G. Kertesz, Jack Tsai, Ann Elizabeth Montgomery

**Affiliations:** 1 Informatics, Decision-Enhancement and Analytic Sciences (IDEAS) Center, Veterans Affairs (VA) Salt Lake City Health Care System, Salt Lake City, Utah; 2 Department of Internal Medicine, The University of Utah School of Medicine, Salt Lake City; 3 VA National Center on Homelessness Among Veterans, Washington, DC; 4 Boston University School of Social Work, Boston, Massachusetts; 5 Center for Healthcare Organization and Implementation Research, Bedford VA Medical Center, Bedford, Massachusetts; 6 Department of Population Health Science, The University of Utah School of Medicine, Salt Lake City; 7 Department of Family Medicine, University of California, Los Angeles, Los Angeles; 8 VA Greater Los Angeles Healthcare System, Los Angeles, California; 9 Birmingham VA Medical Center, Birmingham, Alabama; 10 Department of Medicine, University of Alabama at Birmingham, Birmingham; 11 The University of Texas Health Sciences Center School of Public Health, San Antonio; 12 Department of Health Behavior, University of Alabama at Birmingham School of Public Health, Birmingham

## Abstract

**Importance:**

Temporary financial assistance (TFA) for housing-related expenses is a key component of interventions to prevent homelessness or to quickly house those who have become homeless. Through the US Department of Veterans Affairs (VA) Supportive Services for Veteran Families (SSVF) program, the department provides TFA to veterans in need of housing assistance.

**Objective:**

To assess the association between TFA and housing stability among US veterans enrolled in the SSVF program.

**Design, Setting, and Participants:**

This retrospective cohort study analyzed data on veterans who were enrolled in the SSVF program at 1 of 203 partner organizations in 49 US states and territories. Some veterans had repeat SSVF episodes, but only the first episodes were included in this analysis. An episode was defined as the period between entry into and exit from the program occurring between October 1, 2015, and September 30, 2018.

**Exposures:**

Receipt of TFA.

**Main Outcomes and Measures:**

The main outcome was stable housing, defined as permanent, independent residence with payment by the program client or housing subsidy after exit from the SSVF program. Covariates included demographic characteristics, monthly income and source, public benefits, health insurance, use of other VA programs for homelessness, comorbidities, and geographic location. Multivariable mixed-effects logistic regression, inverse probability of treatment weighting, and instrumental variable approaches were used.

**Results:**

The overall cohort consisted of 41 969 veterans enrolled in the SSVF program, of whom 29 184 (mean [SD] age, 50.4 [12.9] years; 25 396 men [87.0%]) received TFA and 12 785 (mean [SD] age, 50.0 [13.3] years; 11 229 men [87.8%]) did not receive TFA. The mean (SD) duration of SSVF episodes was 90.5 (57.7) days. A total of 69.5% of SSVF episodes involved receipt of TFA, and the mean (SD) amount of TFA was $6070 ($7272). Stable housing was obtained in 81.4% of the episodes. Compared with those who did not receive TFA, veterans who received TFA were significantly more likely to have stable housing outcomes (risk difference, 0.253; 95% CI, 0.240-0.265). An association between the amount of TFA received and stable housing was also found, with risk differences ranging from 0.168 (95% CI, 0.149-0.188) for those who received $0 to $2000 in TFA to 0.226 (95% CI, 0.203-0.249) for those who received more than $2000 to $4000 in TFA.

**Conclusions and Relevance:**

This study found that receipt of TFA through the SSVF program was associated with increased rates of stable housing. These results may inform national policy debates regarding the optimal solutions to prevent and reduce housing instability.

## Key Points

**Question:**

Is temporary financial assistance (TFA) associated with improved housing outcomes among US veterans experiencing housing instability?

**Findings:**

In this cohort study of 41 969 veterans enrolled in the Supportive Services for Veteran Families program, veterans who received TFA were significantly more likely than those who did not receive TFA to exit the program with a stable housing destination.

**Meaning:**

Results of this study suggest that short-term financial assistance for housing-related expenses may be a useful tool for addressing homelessness.

## Teaser

This cohort study examines the association of temporary financial assistance with housing stability among US veterans who are homeless or at risk for homelessness.

## Introduction

Lack of stable housing can have important implications for health and health care utilization. Compared with the general population in the US, homeless individuals have higher rates of infectious diseases (eg, tuberculosis, hepatitis C virus infection, and HIV infection),^1^ age-related comorbidities,^2,3^ poorly controlled chronic conditions,^4,5^ and neuropsychiatric disorders.^6,7,8^ In addition, housing instability has been associated with high rates of mortality^9,10^ among people experiencing long-term^11,12^ or short-term homelessness.^13^ A review^14^ concluded that, outside specific conditions, data have not shown an overall health benefit associated with housing but also noted that housing often serves as the prerequisite to engaging in more regular care. Other studies have reported that housing may be associated with improved physical and mental health outcomes as well as social outcomes, such as fewer encounters with the criminal justice system.^15,16,17,18^

A number of factors are associated with homelessness, including local economic conditions, such as lack of affordable housing and poverty rates,^19^ and personal circumstances, such as financial difficulties,^20,21^ unemployment,^22^ mental illness,^22,23,24^ substance use disorders,^21,23,25^ and lack of health insurance. Programs that provide financial assistance for housing-related expenses with a goal of facilitating housing for previously homeless individuals as quickly as possible may be associated with better health outcomes.

Since October 2011, the US Department of Veterans Affairs (VA) has partnered with community organizations (called *grantees*) to provide housing support and services through the Supportive Services for Veteran Families (SSVF) program. A key component of the SSVF program is temporary financial assistance (TFA), which provides funds for rent, utility bills, security deposit, and other housing-related expenses for veterans who have lost or are at risk of losing stable housing. The goal of housing-related TFA is to prevent homelessness or to quickly house those who have become homeless to prevent more costly interventions later. The SSVF program is described in more detail in the eAppendix in the Supplement. In this study, we assessed the association between TFA and housing stability outcomes among veterans enrolled in the SSVF program.

## Methods

### Study Design and Population

This cohort study used data on veterans enrolled in the SSVF program through grantees throughout the US. We used administrative data from the SSVF program to construct a data set of all SSVF episodes occurring between fiscal years (FYs) October 1, 2015, and September 30, 2018. A veteran’s SSVF episode was defined as the period from the date of enrollment in the SSVF program to the date of program exit. This study was approved by the institutional review board at the University of Utah, which waived informed consent because the research presented no more than minimal risk or harm to participants. This study followed the Strengthening the Reporting of Observational Studies in Epidemiology (STROBE) reporting guideline.^26^

Episode-level TFA data can be unreliable because of the variability in data entry quality across grantees, especially for data from the early period of the SSVF program. However, at the end of each FY, grantees are required to report to the SSVF program office the dollar amounts of TFA (overall and by type of TFA) distributed to veterans during that FY. These end-of-year grantee-level TFA data were available for FYs 2016 to 2018. To ensure that analyses were based on the most reliable episode-level TFA data, we retained only data for episodes that began and ended within the same FY and for grantees in which the sum of TFA dollars provided to individual veterans was no more than 25% different (larger or smaller) from the monetary value of TFA from the end-of-year grantee-level data. This approach accounted for 203 of the 337 grantees (60.2%) between FYs 2016 and 2018. The Figure shows the locations of the SSVF program grantees included in our analysis. Although some veterans had repeated SSVF episodes, we included only the veteran’s first episode in this analysis.

**Figure.**
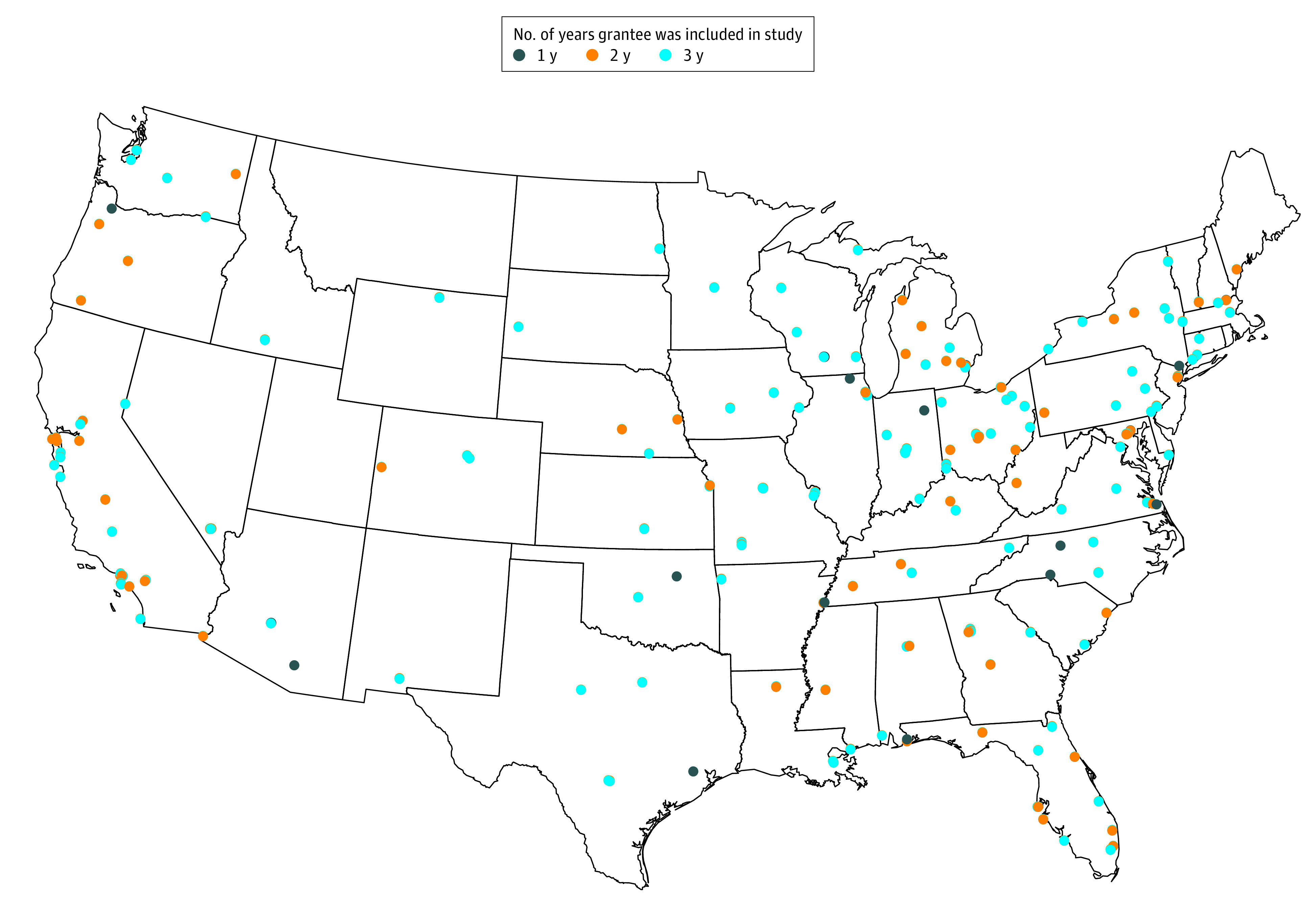
US Locations of the Supportive Services for Veteran Families Program Grantees Included in the Study From Fiscal Year 2016 to Fiscal Year 2018

### Data

The Homeless Management Information System (HMIS) is used to record and store a set of standardized client-level information on characteristics of homeless individuals and the services provided to them through federally funded assistance programs.^27^ We extracted these HMIS data to construct our analytic data set, including episode entry and exit dates, demographic characteristics, employment and educational status, and the type and amount of TFA received through the SSVF program. In addition, we captured enrollment in other VA homeless programs from the Homeless Operations Management and Evaluation System, which tracks homeless veterans as they move through the VA’s homeless programs. We obtained comorbidities data from the VA’s electronic health records stored in the Corporate Data Warehouse, and health care cost data were from the VA Managerial Cost Accounting system. Data from these various sources were linked and were accessed using an identification number unique to each veteran.

### Outcome

The primary outcome was stable housing, defined as permanent, independent residence with payment by the program client or housing subsidy after exit from the SSVF program. We constructed this variable on the basis of a veteran’s housing destination at the end of an SSVF episode as recorded in the HMIS by a case manager. A complete list of exit destinations is provided in eTable 1 in the Supplement.

### Independent Variables

The key independent variables in these analyses were the characteristics of the TFA received by a veteran during an SSVF episode. We characterized TFA as binary (any TFA or no TFA) and as categorical according to the total amount of TFA received during the SSVF episode ($0, >$0 to $2000, >$2000 to $4000, >$4000 to $6000, or >$6000). We created indicators for the type of TFA (ie, rent, security deposit, utilities, moving expenses, other benefits, transportation, and childcare).

Other independent variables were included to reduce confounding in the modeled association between TFA and stable housing. These variables were selected on the basis of previous research that identified factors associated with homelessness.^28,29,30^ Demographic variables included age, sex, presence of a spouse or partner, number of children, and race/ethnicity. Socioeconomic variables included total monthly income, educational level, employment status, homelessness at SSVF program entry, and an indicator for whether the veteran was homeless in the previous 3 years. Indicators for non-TFA services accessed during the SSVF episode included case management, outreach, assistance with VA benefits, assistance with non-VA benefits, direct provision of benefits, and other benefits. Additional variables included indicators for the types of income received, health insurance, and enrollment in other VA homelessness programs. Additional independent variables included the Charlson Comorbidity Index,^31^ mental health diagnoses, VA health care cost in the 365 days before the SSVF episode start date, rurality, distance to the nearest VA medical center, distance to the nearest VA community-based outpatient clinic, and FY of the SSVF episode. We also included the zip code area deprivation index.^32,33^

### Statistical Analysis

We compared the summary measures of independent variables between the TFA and non-TFA recipient groups using a 2-sided *t* test for continuous variables and a 2-sided χ^2^ test for categorical variables. We assessed the association between TFA and the stable housing outcome using 3 different statistical approaches, (the strengths and weaknesses of which are described in the eAppendix in the Supplement.

In our first approach, which was the primary analysis, we fit multivariable mixed-effects logistic regressions with a random effect for grantees to the data, controlling for the aforementioned covariates. As a secondary analysis, we used propensity scores to conduct inverse probability of treatment weighting (IPTW) to balance observed patient characteristics across veterans who received TFA and those who did not receive TFA.^34,35,36^ We calculated the probability of having received TFA using a multivariable logistic regression that accounted for the factors of stable housing as described above.^37^ We then estimated the outcome model using a mixed-effects logistic regression that controlled for covariates (ie, a doubly robust approach).

Although these first 2 statistical approaches decreased the influence of measured confounders, the results could still be biased because of unmeasured confounding. For example, SSVF program grantees could preferentially select veterans for TFA who have more promising housing prospects or who are perceived as easier to house, a practice commonly referred to as *creaming*. We mitigated against bias from creaming in part by controlling for observable characteristics that might be viewed favorably by SSVF programs. However, some of the differences between veterans who did and did not receive TFA were not measured.

Our third statistical approach used an instrumental variable, which can overcome bias from unmeasured confounding in an estimated effect. In this approach, the determination of who received and who did not receive TFA was at the discretion of the grantee, which means that veterans who enrolled in the SSVF program through grantees that allocated TFA more freely than others were more likely to receive TFA. We created 2 summary measures of a grantee’s TFA allocation and used them as instrumental variables: the mean amount of TFA per SSVF episode and the proportion of SSVF episodes in which any amount of TFA was received. We implemented the instrumental variable approach using the 2-stage residual inclusion method given that the outcome model was nonlinear.^38^ As an additional secondary analysis, we assessed the association between the dollar amount and type of TFA received and stable housing outcomes using a multivariable mixed-effects logistic regression model.

Each analysis was run for the overall cohort and then separately as additional secondary analyses for the subsets of veterans for whom the SSVF episode used the rapid rehousing component of the SSVF program (for veterans experiencing homelessness) and for those for whom the episode used the homelessness prevention component of the SSVF program (for veterans at risk for homelessness). The results of each analytic approach are represented as risk differences produced using marginal standardization in which the estimated probability of stable housing was calculated as a weighted mean across each covariate included in the model separately for each level of the exposure variable of interest.^39^ Because of the potential for type I error owing to multiple comparisons, the findings for analyses of secondary and subgroup analyses should be interpreted as exploratory. All statistical analyses were performed using Stata, version 15 (StataCorp LLC) using an a priori statistical significance of a 2-sided *P* = .05.

## Results

Table 1 shows the summary statistics for the overall cohort (N = 41 969) and the subsets of veterans who received TFA during their SSVF episode (n = 29 184; 25 396 male [87.0%]; mean [SD] age, 50.4 [12.9] years) and those who did not receive TFA (n = 12 785; 11 229 male [87.8%]; mean [SD] age, 50.0 [13.3] years). The mean (SD) duration of SSVF program episodes was 90.5 (57.7) days. The eFigure in the Supplement shows the unweighted and weighted standardized differences between TFA and non-TFA recipients for each of the individual characteristics listed in Table 1 after the IPTW analysis. With the weights applied, the standardized difference was below 0.10 for each variable, indicating a high degree of balance.^40^

**Table 1.** Descriptive Statistics of Supportive SSVF Program Enrollees Among Those Who Did and Did Not Receive TFA^a^

Characteristic	Veterans who received TFA (n = 29 184)	Veterans who did not receive TFA (n = 12 785)	*P* value
Age			
Mean (SD), y	50.4 (12.9)	50.0 (13.3)	.003
<40 y	7089 (24.3)	3323 (26.0)	.002
40 to <50 y	4702 (16.1)	2053 (16.1)	
50 to <60 y	9607 (32.9)	4048 (31.7)	
≥60 y	7786 (26.7)	3361 (26.3)	
Sex			
Male	25 396 (87.0)	11 229 (87.8)	.03
Female	3788 (13.0)	1556 (12.2)	
Spouse or partner	5436 (18.6)	2211 (17.3)	<.001
Children	6481 (22.2)	2624 (20.5)	<.001
Race/ethnicity			
White	16 033 (54.9)	7256 (56.8)	<.001
Black	12 834 (44.0)	5268 (41.2)	
Native American	872 (3.0)	519 (4.1)	
Other	497 (1.7)	212 (1.7)	
Total monthly income, US $			
0	8670 (29.7)	4102 (32.1)	<.001
>0 to 500	2841 (9.7)	1168 (9.1)	
>500 to 1500	12 641 (43.3)	5160 (40.4)	
>1500	5032 (17.2)	2355 (18.4)	
Educational level			
Less than high school	14 380 (49.3)	6469 (50.6)	.02
High school diploma	7849 (26.9)	3402 (26.6)	
Some college	4244 (14.5)	1827 (14.3)	
College degree	2711 (9.3)	1087 (8.5)	
Employment status			
No evidence^b^	28 056 (96.1)	12 318 (96.4)	.01
Part-time	336 (1.2)	160 (1.3)	
Full-time	792 (2.7)	307 (2.4)	
Homelessness in past 3 y	11 374 (39.0)	4759 (37.2)	<.001
Income type			
Earned	5145 (17.6)	2208 (17.3)	.37
SSI	3652 (12.5)	1587 (12.4)	.77
VA disability	9159 (31.4)	3808 (29.8)	<.001
Other	533 (1.8)	242 (1.9)	.64
Public benefits			
SNAP	10 872 (37.3)	4351 (34.0)	<.001
Other benefits	1217 (4.2)	485 (3.8)	.07
Health insurance			
Medicaid	4314 (14.8)	2178 (17.0)	<.001
Medicare	2428 (8.3)	1176 (9.2)	.003
VA medical services	22 553 (77.3)	9151 (71.6)	<.001
Employer provided	459 (1.6)	204 (1.6)	.86
Other	1265 (4.3)	622 (4.9)	.02
Type of SSVF program benefits			
Homelessness prevention	9337 (32.0)	3849 (30.1)	<.001
Rapid rehousing	18 346 (62.9)	8337 (65.2)	
Both or missing	1501 (5.1)	599 (4.7)	
Homeless programs			
HUD-VASH	6089 (20.9)	1434 (11.2)	<.001
GPD	3235 (11.1)	1400 (11.0)	.69
Other	4377 (15.0)	1739 (13.6)	<.001
Charlson Comorbidity Index, mean (SD)	1.0 (2.0)	0.9 (1.9)	<.001
Mental health diagnosis	15 973 (54.7)	6618 (51.8)	<.001
VA cost in 365 d before SSVF program entry date, mean (SD), US $			
Outpatient	9256 (12 833)	7620 (11 163)	<.001
Inpatient	7041 (27 387)	6759 (25 880)	.84
ADI^c^			
<44	5137 (17.6)	2142 (16.8)	<.001
44 to <60	5100 (17.5)	1921 (15.0)	
60 to <73	5347 (18.3)	2084 (16.3)	
≥73	5795 (19.9)	2025 (15.8)	
Missing	7805 (26.7)	4613 (36.1)	
Rurality	3251 (11.1)	1419 (11.1)	.90
Fiscal year of the SSVF episode			
2016	12 198 (41.8)	5321 (41.6)	.67
2017	11 183 (38.3)	4955 (38.8)	
2018	5803 (19.9)	2509 (19.6)	

Abbreviations: ADI, area deprivation index; GPD, Grant and Per Diem; HUD-VASH, US Department of Housing and Urban Development-VA Supportive Housing; SNAP, Supplemental Nutrition Assistance Program; SSI, Supplemental Security Income; SSVF, Supportive Services for Veteran Families; TFA, temporary financial assistance; VA, US Department of Veterans Affairs.

^a^ Data are presented as number (percentage) of veterans unless otherwise indicated.

^b^ No evidence of employment was recorded at the time of enrollment in the SSVF program.

^c^ Higher numbers indicate less disadvantage.

The percentages of veterans who obtained stable housing by the amount of TFA received are shown in Table 2. Stable housing was obtained in 81.4% of the episodes. An association between the amount of TFA received and stable housing was found, with risk differences ranging from 0.168 (95% CI, 0.149-0.188) for those who received $0 to $2000 in TFA to 0.226 (95% CI, 0.203-0.249) for those who received more than $2000 to $4000 in TFA. More than 90% of veterans in both rapid rehousing and homelessness prevention components with TFA amounts of at least $2000 exited the program to stable housing. Stable housing rates were higher for veterans enrolled in homelessness prevention compared with rapid rehousing for both those who did not receive TFA (3160 [82.1%] vs 4103 [49.2%]) and those who received more than $0 to $2000 of TFA (2067 [94.0%] vs 3390 [77.7%]). A total of 69.5% of SSVF episodes involved the receipt of TFA, and the mean (SD) amount of TFA was $6070 ($7272).

**Table 2.** Unadjusted Percentage of Veterans Obtaining Stable Housing After Exit From the Supportive Services for Veteran Families Program

Amount of TFA, $	Overall		Rapid rehousing only		Homelessness prevention only	
	Total veterans, No.	Veterans obtaining stable housing, No. (%)	Total veterans, No.	Veterans obtaining stable housing, No. (%)	Total veterans. No.	Veterans obtaining stable housing, No. (%)
>0	29 184	26 782 (91.8)	18 346	16 505 (90.0)	9337	8926 (95.6)
0	12 785	7564 (59.2)	8337	4103 (49.2)	3849	3160 (82.1)
>0 to 2000	7048	5847 (83.0)	4365	3390 (77.7)	2199	2067 (94.0)
>2000 to 4000	7284	6913 (94.9)	4392	4137 (94.2)	2490	2397 (96.3)
>4000 to 6000	4956	4681 (94.5)	3185	2988 (93.8)	1551	1485 (95.7)
>6000	9896	9341 (94.4)	6404	5990 (93.5)	3097	2977 (96.1)

Abbreviation: TFA, temporary financial assistance.

In multivariable regression analyses (Table 3; unadjusted results shown in eTable 2 in the Supplement), veterans who received any amount of TFA were significantly more likely to have a stable housing outcome compared with those who did not receive TFA (risk difference, 0.253; 95% CI, 0.240-0.265). This association was stronger for those enrolled in the rapid rehousing component (risk difference, 0.301; 95% CI, 0.288-0.315) compared with those in the homelessness prevention component (risk difference, 0.112; 95% CI, 0.097-0.127). The IPTW analysis yielded similar results, with a significant increase in the probability of stable housing for those who received TFA compared with those who did not. We also found an association between TFA and stable housing using the instrumental variable approach, with risk differences ranging from 0.077 (95% CI, 0.021-0.133) to 0.119 (95% CI, 0.070-0.169) for rapid rehousing and from 0.037 (95% CI, 0.005-0.069) to 0.042 (95% CI, 0.008-0.076) for homelessness prevention. The *F* statistic from the instrumental variable models ranged from 74.20 to 195.91, all of which are considerably higher than 10, the generally accepted threshold for the instrument to be sufficiently strong for use in an instrumental variable analysis.^41^

**Table 3.** Association of Receipt of TFA With Stable Housing

Analytical approach	Overall		Rapid rehousing only		Homelessness prevention only	
	Risk difference (95% CI)	*P* value	Risk difference (95% CI)	*P* value	Risk difference (95% CI)	*P* value
Multivariable regression^a^	0.253 (0.240-0.265)	<.001	0.301 (0.288-0.315)	<.001	0.112 (0.097-0.127)	<.001
Inverse probability of treatment weighting	0.314 (0.287-0.341)	<.001	0.365 (0.338-0.392)	<.001	0.142 (0.104-0.180)	<.001
Instrumental variable						
Mean amount of TFA per SSVF episode	0.061 (0.018-0.104)	.006	0.077 (0.021-0.133)	.007	0.042 (0.008-0.076)	.02
Proportion of SSVF episodes with any receipt of TFA	0.095 (0.057-0.132)	<.001	0.117 (0.067-0.166)	<.001	0.037 (0.004-0.069)	.03
Both	0.096 (0.058-0.133)	<.001	0.119 (0.070-0.169)	<.001	0.037 (0.005-0.069)	.03

Abbreviations: SSVF, Supportive Services for Veteran Families; TFA, temporary financial assistance; VA, US Department of Veterans Affairs.

^a^ Multivariable mixed-effects logistic regression models included the following covariates: demographic variables (age, sex, presence of spouse or partner, number of children, and race/ethnicity); socioeconomic status (total monthly income, educational level, employment status, and number of times the veteran was homeless in the previous 3 years); type of income (earned, unemployment, Supplemental Security Income, VA disability service-connected, VA disability non–service-connected, private disability, and workers’ compensation); indicators for publicly funded benefit programs (Supplemental Nutrition Assistance Program; Women, Infants, and Children; Temporary Aid for Needy Families; and other benefits); type of health insurance (Medicaid, Medicare, State Children’s Health Insurance Program, VA health care, employer-provided insurance, Consolidated Omnibus Budget Reconciliation Act insurance, private pay, state insurance, Indian insurance, and other health insurance); indicators for enrollment in other VA homeless programs (US Department of Housing and Urban Development-VA Supportive Housing vouchers, Grant and Per Diem Program, Compensated Work Therapy, Domiciliary Care for Homeless Veterans, Healthcare for Homeless Veterans [HCHV] Contract Emergency Residential Services Program, HCHV Low Demand Safe Haven, HCHV Case Management Program, Health Care Re-Entry Veterans Program, and Veterans Justice Outreach Program); Charlson Comorbidity Index; VA health care cost in the 365 days prior to the SSVF program entry date; rurality; distance to the nearest VA medical center; distance to the nearest VA community-based outpatient clinic; fiscal year of the SSVF episode; and zip code area deprivation index.

When considering the association between the dollar amount of TFA and stable housing rates (multivariable results shown in Table 4; univariable results shown in eTable 3 in the Supplement), receipt of TFA from more than $0 to $2000 compared with no TFA among those in the rapid rehousing component was associated with a risk difference of 0.198 (95% CI, 0.171-0.225). However, the magnitude of the association was similar for TFAs of more than $2000 to $4000 (risk difference, 0.281; 95% CI, 0.250-0.311), more than $4000 to $6000 (risk difference, 0.269; 95% CI, 0.236-0.302), or more than $6000 (risk difference, 0.269; 95% CI, 0.235-0.304). For the homelessness prevention component, the size of the association of TFA amount with stable housing outcomes increased from 8.0% (95% CI, 5.4%-10.5%) for more than $0 to $2000 to 9.2% (95% CI, 6.1%-12.2%) for more than $6000.

**Table 4.** Multivariable Regression Results of the Association Between the Amount of TFA and Stable Housing Outcome^a^

SSVF program assistance	Overall		Rapid rehousing only		Homelessness prevention only	
	Risk difference (95% CI)	*P* value	Risk difference (95% CI)	*P* value	Risk difference (95% CI)	*P* value
Total amount of TFA, $						
0	1 [Reference]		1 [Reference]		1 [Reference]	
>0 to 2000	0.168 (0.149 to 0.188)	<.001	0.198 (0.171 to 0.225)	<.001	0.080 (0.054 to 0.105)	<.001
>2000 to 4000	0.226 (0.203 to 0.249)	<.001	0.281 (0.250 to 0.311)	<.001	0.091 (0.063 to 0.119)	<.001
>4000 to 6000	0.217 (0.193 to 0.242)	<.001	0.269 (0.236 to 0.302)	<.001	0.086 (0.056 to 0.117)	<.001
>6000	0.219 (0.194 to 0.244)	<.001	0.269 (0.235 to 0.304)	<.001	0.092 (0.061 to 0.122)	<.001
Type of TFA						
Rent	0.041 (0.029 to 0.053)	<.001	0.030 (0.014 to 0.045)	<.001	0.043 (0.025 to 0.062)	<.001
Security deposit	0.126 (0.114 to 0.137)	<.001	0.153 (0.138 to 0.168)	<.001	0.013 (0.002 to 0.036)	.03
Utilities	0.060 (0.047 to 0.073)	<.001	0.069 (0.052 to 0.087)	<.01	0.022 (0.006 to 0.039)	.008
Other benefits	−0.075 (−0.086 to −0.064)	<.001	−0.082 (−0.097 to −0.067)	<.001	−0.050 (−0.066 to −0.034)	<.001
Non-TFA services						
Case management	−0.008 (−0.020 to 0.004)	.20	−0.012 (−0.028 to 0.004)	.13	−0.004 (−0.021 to 0.013)	.65
Outreach	−0.024 (−0.037 to −0.010)	.001	−0.026 (−0.043 to −0.009)	.003	−0.011 (−0.031 to 0.008)	.24
Assistance						
With VA benefits	−0.011 (−0.029 to 0.007)	.22	−0.011 (−0.033 to 0.011)	.35	0.006 (−0.028 to 0.040)	.71
With non-VA benefits	−0.007 (−0.023 to 0.008)	.36	−0.004 (−0.023 to 0.016)	.73	−0.020 (−0.047 to 0.007)	.14
Direct provision of benefits	0.001 (−0.015 to 0.017)	.89	0.004 (−0.016 to 0.024)	.71	0.015 (−0.013 to 0.042)	.29
Other benefits	0.004 (−0.015 to 0.023)	.70	0.006 (−0.020 to 0.032)	.65	0.003 (−0.021 to 0.028)	.79

Abbreviations: HCHV, Healthcare for Homeless Veterans; SSVF, Supportive Services for Veteran Families; TFA, temporary financial assistance; VA, US Department of Veterans Affairs.

^a^ Multivariable mixed-effects logistic regression models included the following covariates: demographic variables (age, sex, presence of spouse or partner, number of children, and race/ethnicity); socioeconomic status (total monthly income, educational level, employment status, and number of times the veteran was homeless in the previous 3 years); type of income (earned, unemployment, Supplemental Security Income, VA disability service-connected, VA disability non–service-connected, private disability, and workers’ compensation); indicators for publicly funded benefit programs (Supplemental Nutrition Assistance Program; Women, Infants, and Children; Temporary Aid for Needy Families; and other benefits); type of health insurance (Medicaid, Medicare, State Children’s Health Insurance Program, VA health care, employer-provided insurance, Consolidated Omnibus Budget Reconciliation Act insurance, private pay, state insurance, Indian insurance, and other health insurance); indicators for enrollment in other VA homeless programs (US Department of Housing and Urban Development-VA Supportive Housing vouchers, Grant and Per Diem Program, Compensated Work Therapy, Domiciliary Care for Homeless Veterans, HCHV Contract Emergency Residential Services Program, HCHV Low Demand Safe Haven, HCHV Case Management Program, Health Care Re-Entry Veterans Program, and Veterans Justice Outreach Program); Charlson Comorbidity Index; VA health care cost in the 365 days prior to the SSVF program entry date; rurality; distance to the nearest VA medical center; distance to the nearest VA community-based outpatient clinic; fiscal year of the SSVF episode; and zip code area deprivation index.

## Discussion

In this study, SSVF program enrollees who received TFA were significantly more likely to have stable housing after exit from the program than were those who did not receive TFA. The magnitude of the association of TFA with stable housing was largest for security deposit TFA among those in the rapid rehousing component and for rent TFA among those in the homelessness prevention component of the SSVF program. One possible explanation for this finding may be that veterans in the rapid rehousing and homelessness prevention components experienced different types of housing challenges. For example, the up-front fixed cost of a security deposit may be difficult to obtain for someone who is struggling financially and is currently homeless. On the other hand, obtaining money for a security deposit may not be the most daunting challenge for those who are currently housed but are at risk of becoming homeless. For these individuals, financial assistance to pay rent to maintain their housing may be more useful. The different types of TFA appear to target veterans with different housing assistance needs.

It is important to place these results in the context of previous studies of nonveteran populations. Three quasi-experimental studies found that rapid rehousing was associated with a decrease in returns to an emergency shelter.^42,43,44^ The Family Options Study^45,46^ was a large randomized clinical trial of rapid rehousing compared with 3 alternatives: usual care, transitional housing, and permanent housing subsidy. At both 20 months^45^ and 37 months,^46^ housing outcomes for rapid rehousing were no different from outcomes for usual care or transitional housing but were worse than outcomes for permanent housing subsidy, a more robust form of intervention. A randomized clinical trial^47^ that focused on individuals with HIV infection or AIDS found that individuals in the rapid rehousing intervention group were more likely to be placed in stable housing than were those receiving usual care.

We believe this innovative assessment of the association between TFA and stable housing is relevant to policy makers given the increasing emphasis in federal homeless policy over the past decade on rapid rehousing programs that, similar to the SSVF program, provide TFA.^48,49^ For example, between 2013 and 2019, the availability of rapid rehousing interventions increased by nearly 5-fold.^50^ Given the high cost of providing services to homeless individuals and the substantial adverse implications of homelessness for both physical and mental health, the primary goal of any rapid rehousing program is to facilitate stable housing. From this perspective, the results of this cohort study may support a continued and perhaps expanded policy shift toward offering this type of assistance to a larger number of households that are experiencing homelessness.

The small number of high-quality research studies of rapid rehousing programs highlights the scarcity of research in this area, and studies focused on homelessness prevention are fewer still. One study of homelessness prevention analyzed calls between 2010 and 2012 to the Homelessness Prevention Call Center in Chicago from individuals at imminent risk of eviction requesting TFA that would allow them to remain in their home.^51^ The study found that receiving TFA was significantly associated with a decreased likelihood that a caller was admitted to a homeless shelter and with a decrease in the number of days spent in a shelter.^51^ The results of the present study are broadly consistent with these previous findings.

### Strengths and Limitations

This study has strengths. First, the use of detailed HMIS and VA clinical data allowed the inclusion of a rich set of individual covariates in the statistical models. Although the TFA exposure was not randomly assigned in this study, these covariates allowed us to achieve a high level of conditional exchangeability between the SSVF program clients who received or did not receive TFA.^52^ Second, we found consistent results across the 3 different estimation approaches: multivariable regression, IPTW, and instrumental variable. Third, identifying a suitable control group can be difficult when studying an intervention retrospectively, but for this study, the control group was composed of veterans who also enrolled in the SSVF program; thus, they were facing similar housing instability problems as those who received TFA. In addition, the SSVF program entry date provided a natural and consistent index date for both the intervention and the control groups. Fourth, other studies on the association of housing interventions with stable housing outcomes have focused on limited geographic areas. However, the present study included veterans from 203 grantees across 49 US states and territories, making it one of the most geographically expansive studies conducted on this topic.

This study also has limitations. First, because the study focused on the US veteran population, the results may not be generalizable to other groups of homeless individuals. Second, the stable housing outcome was measured at exit from the SSVF program, with episodes lasting a mean of 90.5 days. We were, therefore, able to draw conclusions only about the association between TFA and short-term housing stability. Third, although the HMIS is a rich source of data, the information contained in this database is self-reported by program clients. Fourth, even though the HMIS and VA electronic data allowed us to control for a number of important confounders in the association between TFA and stable housing, it was impossible to capture all of the factors that would influence a grantee’s decision to allocate TFA to a veteran. For this reason, the estimates from the multivariable regression and IPTW analyses may still be biased because of confounding by indication.

## Conclusions

The findings of this cohort study suggest that receipt of TFA through the SSVF program may be associated with increased rates of stable housing among US veterans. These results may inform national policy debates regarding the optimal solutions to housing instability.

## Notes

Supplement. **eAppendix.** **eTable 1.** Exit Destinations and Classifications **eFigure.** Weighted and Unweighted Standardized Differences Between TFA and Non-TFA Recipients **eTable 2.** Univariable Regression Results Relating Amount of Temporary Financial Assistance (TFA) to Stable Housing Outcome **eTable 3.** Unadjusted Effect of Any Temporary Financial Assistance (TFA) on Stable Housing
