# Supplementary material for: Association of Temporary Financial Assistance With Housing Stability Among US Veterans in the Supportive Services for Veteran Families Program
Source: JAMA Netw Open. 2021 Feb 10;4(2):e2037047. doi: 10.1001/jamanetworkopen.2020.37047 (PMC8015862; doi:10.1001/jamanetworkopen.2020.37047)
Supplement: Supplement. — eAppendix. eTable 1. Exit Destinations and Classifications eFigure. Weighted and Unweighted Standardized Differences Between TFA and Non-TFA Recipients eTable 2. Univariable Regression Results Relating Amount of Temporary Financial Assistance (TFA) to Stable Housing Outcome eTable 3. Unadjusted Effect of Any Temporary Financial Assistance (TFA) on Stable Housing [file jamanetwopen-e2037047-s001.pdf]

Supplementary Online Content

Nelson RE, Byrne TH, Suo Y, et al. Association of temporary financial assistance with housing stability among US veterans in the Supportive Services for Veteran Families program. *JAMA Netw Open*. 2021;4(2): e2037047. doi:10.1001/jamanetworkopen.2020.37047

eAppendix.
eTable 1. Exit Destinations and Classifications
eFigure. Weighted and Unweighted Standardized Differences Between TFA and Non-TFA Recipients
eTable 2. Univariable Regression Results Relating Amount of Temporary Financial Assistance (TFA) to Stable Housing Outcome
eTable 3. Unadjusted Effect of Any Temporary Financial Assistance (TFA) on Stable Housing

This supplementary material has been provided by the authors to give readers additional information about their work.

©2021 Nelson RE et al. *JAMA Network Open*

eAppendix

Overview of SSVF program

Through the SSVF program, the VA awards grants to community-based non-profit organizations to assist Veterans facing housing instability. The SSVF program has two components: rapid re-housing (RRH), which serves Veterans who no longer have stable housing, and homelessness prevention (HP), which serves Veterans who are at an imminent risk of losing stable housing.

All SSVF grantees are required to provide the following services: 1) outreach services (within the community and the VA), 2) case management (including needs assessment and a housing stability plan), 3) assistance in obtaining VA benefits, 4) assistance in obtaining non-VA benefits (e.g., legal assistance, credit counseling, and income support such as Temporary Assistance for Needy Families (TANF) and Supplemental Nutrition Assistance Program (SNAP)), and 5) TFA, including assistance with rent, utility payments, security deposits, moving expenses, child care, and transportation.

Since its inception, the SSVF program has grown from 85 grantees in 40 states and the District of Columbia in FY2012 to 308 grantees in all 50 states in FY2018. During this time, total expenditures rose from $59 million to $333 million annually and the number of households served increased from 21,111 to 84,411 annually with cumulatively more than 500,000 households receiving benefits over a period of time between FY2012 and FY2018.

Strengths and weaknesses of analytical approaches

Because our study was not a randomized controlled trial, the estimated relationship of interest (between TFA and stable housing) is subject to confounding bias. We used 3 different approaches to reduce this bias. The first was to include measures of confounders as covariates in a multivariable regression model. The benefit of this approach is that it is commonly used and therefore easily understandable for most readers. By controlling for measured confounders in a multivariable regression model, the effect of the exposure of interest on the outcome is isolated. While regression is a powerful statistical tool, an important limitation is that it only overcomes bias from *measured* confounders. In other words, even in multivariable regression models, unmeasured confounding could still bias the effect estimates.

Our second approach was inverse probability of treatment weighting (IPTW). In this approach, a propensity score (PS) is estimated by performing a logistic regression of the treatment variable – TFA, in our case – on all other independent variables. The PS is defined as the probability of receiving treatment, given a set of covariates. Once the propensity score is calculated, it can be used to reweight individuals in the analysis to increase the weight for those receiving unexpected exposures (i.e., those who, based on the value of their observable covariates had a high propensity score for treatment but were, in fact, untreated or vice versa). This exercise produces a pseudo-population of individuals in whom covariates are balanced between treatment groups, thus, overcoming confounding bias due to measured confounders. These weights are calculated as 1/PS for patients in the treated group and [1/(1-PS)] for patients in the control group. As with multivariable adjustment, IPTW can overcome confounding bias due to measured confounders but not due to unmeasured confounders.

©2021 Nelson RE et al. *JAMA Network Open*

Our final analytical approach was instrumental variables (IV). This approach overcomes confounding bias due to unmeasured confounders through a variable which (1) influences the treatment variable but (2) does not influence the outcome other than through its influence on the treatment variable. It is important that the effect of this IV on the treatment variable is sufficiently strong, so as to induce enough variation in the treatment variable to allow for estimation of its effect on the outcome. The ability of the IV approach to overcome unmeasured confounding is a clear advantage of this estimation approach. However, there are many important disadvantages as well. For instance, the ability of an IV to overcome unmeasured confounding relies on it being a valid instrument through the 2 criteria outlined above. In practice, it can be difficult to identify a variable that fulfills both of these requirements. Another important caveat to keep in mind is that, while multivariable regression and IPTW yield average treatment effects (ATEs), an IV estimation approach yields a local average treatment effect (LATE). When a treatment is assigned to a certain group of patients, those patients may or may not choose to take up that treatment. This creates 4 potential groups of patients: (i) compliers, those who take the treatment when it is assigned to them but not if it is not assigned to them, (ii) always-takers, those who take the treatment regardless of whether it was assigned to them or not, (iii) never-takers, those who never take the treatment regardless of whether it was assigned to them or not, and (iv) defiers, those who take the treatment when it is not assigned to them but do not take it if it is not assigned to them. The LATE is the treatment effect in the compliers group only. In other words, the IV approach does not estimate the effect of the treatment in the other 3 groups. In our case of TFA within the SSVF program, this means that our IV approach estimates the effect of TFA on individuals whose receipt of TFA depends on the grantee through which they access the SSVF program. When the IV was specified as the proportion of clients receiving TFA, these compliers are Veterans who would have been more likely to receive TFA had they accessed the SSVF program through a grantee that gave a relatively high proportion of clients TFA but less likely to receive TFA if they had accessed the program through a grantee that gave a relatively low proportion of clients TFA. The LATE is different for each IV, which is why it was important for us to try several IVs. Finally, the LATE may be similar to the ATE if the share of the compliers is large or if the treatment effects for each of the 4 groups of individuals are similar.

©2021 Nelson RE et al. *JAMA Network Open*

eTable 1. Exit Destinations and Classifications

Exit destination	Classified as stable housing?
Emergency shelter, including hotel or motel paid for with emergency shelter voucher	No
Transitional housing for homeless persons (including homeless youth)	No
Permanent housing (other than RRH) for formerly homeless persons	Yes
Psychiatric hospital or other psychiatric facility	No
Substance abuse treatment facility or detox center	No
Hospital or other residential non-psychiatric medical facility	No
Jail, prison or juvenile detention facility	No
Client doesn’t know	No
Client refused	No
Rental by client, no ongoing housing subsidy	Yes
Owned by client, no ongoing housing subsidy	Yes
Staying or living with family, temporary tenure (e.g., room, apartment or house)	No
Staying or living with friends, temporary tenure (e.g., room apartment or house)	No
Hotel or motel paid for without emergency shelter voucher	No
Foster care home or foster care group home	No
Place not meant for habitation (e.g., a vehicle, an abandoned building, bus/train/subway station/airport or anywhere outside)	No
Other	No
Safe Haven	No
Rental by client, with VASH housing subsidy	Yes
Rental by client, with other ongoing housing subsidy	Yes
Owned by client, with ongoing housing subsidy	Yes
Staying or living with family, permanent tenure	Yes
Staying or living with friends, permanent tenure	Yes
Deceased	No
Long-term care facility or nursing home	No
Moved from one HOPWA funded project to HOPWA PH	No
Moved from one HOPWA funded project to HOPWA TH	No
Rental by client, with GPD TIP housing subsidy	Yes
Residential project or halfway house with no homeless criteria	No
No exit interview completed	No
Rental by client, with RRH or equivalent subsidy	Yes
Data not collected	No

©2021 Nelson RE et al. *JAMA Network Open*

eFigure. Weighted and Unweighted Standardized Differences Between TFA and Non-TFA Recipients

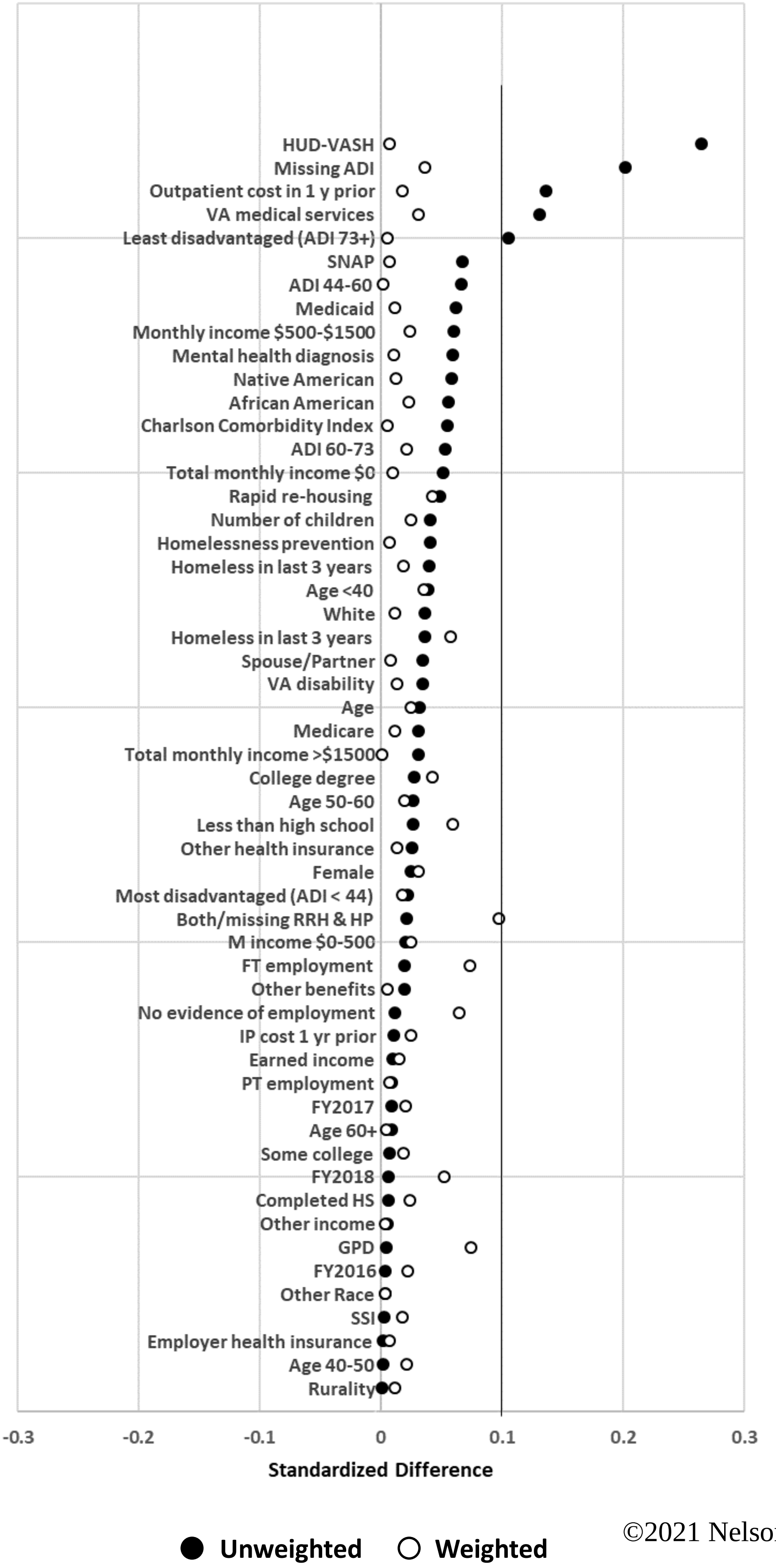

©2021 Nelson RE et al. *JAMA Network Open*

eTable 2. Univariable Regression Results Relating Amount of Temporary Financial Assistance (TFA) to Stable Housing Outcome

	Overall				Rapid Re-Housing Only				Homelessness Prevention Only			
			95% CI				95% CI				95% CI	
**SSVF benefits**	**Risk difference**	**P-value**	**LL**	**UL**	**Risk difference**	**P-value**	**LL**	**UL**	**Risk difference**	**P-value**	**LL**	**UL**
Total amount of TFA (ref = $0)												
$0-$2,000	0.260	<0.001	0.237	0.283	0.237	<0.001	0.209	0.265	0.098	<0.001	0.070	0.127
$2,000-$4,000	0.324	<0.001	0.298	0.351	0.323	<0.001	0.291	0.354	0.111	<0.001	0.081	0.142
$4,000-$6,000	0.317	<0.001	0.289	0.344	0.310	<0.001	0.276	0.344	0.104	<0.001	0.072	0.137
$6,000+	0.320	<0.001	0.292	0.348	0.308	<0.001	0.274	0.343	0.110	<0.001	0.077	0.143
Type of TFA												
Rent	0.033	<0.001	0.020	0.045	-0.010	0.210	-0.025	0.005	0.036	<0.001	0.017	0.055
Security deposit	0.111	<0.001	0.099	0.123	0.180	<0.001	0.165	0.195	0.018	0.044	0.001	0.035
Utilities	0.081	<0.001	0.067	0.095	0.085	<0.001	0.067	0.103	0.025	0.003	0.009	0.042
Other benefits	-0.137	<0.001	-0.149	-0.124	-0.103	<0.001	-0.119	-0.087	-0.056	<0.001	-0.072	-0.040

©2021 Nelson RE et al. *JAMA Network Open*

eTable 3. Unadjusted Effect of Any Temporary Financial Assistance (TFA) on Stable Housing

	Overall				Rapid Re-Housing Only				Homelessness Prevention Only			
			95% CI				95% CI				95% CI	
**Analytic approach**	**Risk difference**	**P-value**	**LL**	**UL**	**Risk difference**	**P-value**	**LL**	**UL**	**Risk difference**	**P-value**	**LL**	**UL**
Univariable regression	0.281	<0.001	0.267	0.295	0.322	<0.001	0.308	0.337	0.118	<0.001	0.102	0.133
Inverse probability of treatment weights	0.322	<0.001	0.290	0.353	0.363	<0.001	0.334	0.392	0.146	<0.001	0.104	0.188
Instrumental variable (IV)												
IV = Mean amount of TFA per SSVF episode	0.564	<0.001	0.522	0.606	0.506	<0.001	0.467	0.544	0.090	<0.001	0.060	0.121
IV = Proportion of SSVF episodes receiving any TFA	0.506	<0.001	0.468	0.543	0.474	<0.001	0.438	0.510	0.084	<0.001	0.055	0.113
IV = Both instrumental variables	0.506	<0.001	0.469	0.543	0.475	<0.001	0.439	0.511	0.084	<0.001	0.055	0.113

©2021 Nelson RE et al. *JAMA Network Open*
